# Deimatic Display in the European Swallowtail Butterfly as a Secondary Defence against Attacks from Great Tits

**DOI:** 10.1371/journal.pone.0047092

**Published:** 2012-10-08

**Authors:** Martin Olofsson, Stephan Eriksson, Sven Jakobsson, Christer Wiklund

**Affiliations:** Department of Zoology, Stockholm University, Stockholm, Sweden; University of Sussex, United Kingdom

## Abstract

**Background:**

Many animals reduce the risk of being attacked by a predator through crypsis, masquerade or, alternatively, by advertising unprofitability by means of aposematic signalling. Behavioural attributes in prey employed after discovery, however, signify the importance of also having an effective secondary defence if a predator uncovers, or is immune to, the prey’s primary defence. In butterflies, as in most animals, secondary defence generally consists of escape flights. However, some butterfly species have evolved other means of secondary defence such as deimatic displays/startle displays. The European swallowtail, *Papilio machaon*, employs what appears to be a startle display by exposing its brightly coloured dorsal wing surface upon disturbance and, if the disturbance continues, by intermittently protracting and relaxing its wing muscles generating a jerky motion of the wings. This display appears directed towards predators but whether it is effective in intimidating predators so that they refrain from attacks has never been tested experimentally.

**Methodology/Principal Findings:**

In this study we staged encounters between a passerine predator, the great tit, *Parus major*, and live and dead swallowtail butterflies in a two-choice experiment. Results showed that the dead butterfly was virtually always attacked before the live butterfly, and that it took four times longer before a bird attacked the live butterfly. When the live butterfly was approached by a bird this generally elicited the butterfly’s startle display, which usually caused the approaching bird to flee. We also performed a palatability test of the butterflies and results show that the great tits seemed to find them palatable.

**Conclusions/Significance:**

We conclude that the swallowtail’s startle display of conspicuous coloration and jerky movements is an efficient secondary defence against small passerines. We also discuss under what conditions predator-prey systems are likely to aid the evolution of deimatic behaviours in harmless and palatable prey.

## Introduction

Most species are subjected to predation as evidenced by ubiquitous morphological and behavioural traits that have evolved to minimise the risk of being attacked and killed. According to Edmunds [1] these defences operate either to prevent predator attacks from taking place (i.e. primary defence) or to prevent predator attacks from being successful once they have been initiated (i.e. secondary defence). Essentially, primary defence either reduces the likelihood of prey detection through crypsis [2], [3], or recognition through masquerade [4] or conversely alerts the attention of predators by aposematic signalling of unprofitability [5]. Secondary defence includes a variety of responses such as evasion, deflection of attacks and death feigning [1], [6]. Another defence that several prey species employ when attacked by predators is startle displays (or ‘deimatic behaviour’) which typically consist of a sudden exposure of conspicuous colours and patterns which are often accompanied by other stimuli such as sounds [1]. Essentially, prey employing deimatic behaviours can be divided into species that signal honest harmful properties, such as toxicity or an ability to retaliate, or species that signal deceitfully inasmuch they lack means to harm the predator [1]. A third, albeit functionally different, form of secondary defence display, called ‘pursuit-deterrence’, may aid survival by advertising flight potency or that the prey is difficult to handle and so reduces the willingness of the predator to attempt to attack the prey [7].

A study set out to investigate the efficiency and function of a putative anti-predator behaviour should benefit from focusing on the predator since its reaction to the prey’s behaviour is likely to reflect the underlying evolutionary predicates for a certain defence to evolve, cf. [8]. For example, when predators perform an escape flight as a response to a displaying prey, it seems reasonable to assume that the prey animal’s defence ultimately capitalizes on the predator’s risk of making an ‘acceptance error’, cf. [9]; that is, approaching and attacking one of its own predators or a dangerous prey, which could be fatal to the predator [10]–[13].

In a recent study, Vallin and colleagues [14] investigated the diversity of defences in three closely related nymphalid butterfly species, the peacock butterfly (*Inachis io*), the small tortoiseshell (*Aglais urticae*) and the comma (*Polygonia c-album*) [15]. The principal conclusion of this study was that despite close relatedness, similar ecology and, most likely, shared predators, peacocks and commas in particular have evolved different anti-predation tactics. Both species share the same primary defence allegedly mimicking leafs when resting [16], [17] but diverge distinctly in their behaviours during an encounter with a predator. Vallin and colleagues showed that the peacocks survived encounters with blue tits, *Cyanistes caeruleus*, by suddenly revealing their dorsal wing surface as soon as the bird entered the close vicinity of the butterfly, thereby exposing four eyespots which intimidated the birds so that they refrained from attacking [18], [19]. In contrast, the commas, that appear to masquerade as leaves, never moved no matter how close the bird approached and survived presumably because they were not recognized as insect prey by the birds, cf. [4].

Wing-flicking as a secondary defence in butterflies is, however, not only found in peacocks (and other related Nymphalids); the European swallowtail (*Papilio machaon*), which belongs to the butterfly family Papilionidae, exhibits a similar behaviour [20]. When at rest the European swallowtail keeps its wings closed above the body (identical to the resting posture of the peacock) and upon disturbance flicks its wings open, similar to the behaviour displayed by peacocks. The peacock has two modes of wing-flicking: it may (i) open and close its wings in a repeated sequence or (ii) open its wings suddenly and thereafter keeping them constantly exposed showing the dorsal wing surface and simultaneously protracting and relaxing the wings in a pulsating sequence [18], [19]. The European swallowtail does not open and close its wings in a sequence; its behaviour is instead similar to the latter behaviour displayed by the distantly related peacock (this study). Wiklund and Sillén-Tullberg [20] tested survival of larvae, pupae and adults of the European swallowtail when facing attacks from a group of Japanese quails (*Coturnix coturnix japonica*). One result of their study was that none of the eight tested adult swallowtails were killed by the birds, despite the fact that half of them were seized. The experimenters in this study never tested the palatability of the butterflies to the birds (swallowtails were assumed to be aposematic) and suggested that the defensive behaviour (i.e. ‘wing fluttering’ or wing flicking) was instrumental in aiding the survival of the butterflies since the birds often fled from the displaying butterfly. Furthermore, the European swallowtail is interesting from the perspective that it lacks eyespots, and only displays strong contrasting colours of black and yellow when disturbed. Although the literature provides many examples of prey animals that employ eyespot-less but conspicuous colour displays, like that of the European swallowtail [1], [3], [6], [21], empirical evidence of an anti-predator effect of such displays has rarely been documented [6]. One classical example comprises palatable noctuid moths of the genus *Catocala* which are cryptic when resting, but may suddenly reveal their hind wings and so exposing colours such as red, yellow and blue, often contrasted with black patterns [6], [22]. Experiments using artificial models of these moths indicate that conspicuousness, novelty, oddity and anomaly are instrumental features of an effective startle display when targeted on avian predators [23].

In this study, we staged encounters between a passerine predator, the great tit, *Parus major*, and live versus dead European swallowtail butterflies in a two-choice experiment. Our objective was to elucidate whether the behavioural attribute of wing-flicking is important to protect the butterfly from bird predation or whether the conspicuous coloration *per se* could intimidate a bird predator. We also assessed the birds’ reaction to the butterflies’ wing-flicking and the palatability of European swallowtails to great tits by offering freshly killed butterflies to the birds in their home cages.

## Materials and Methods

### (a) Study Species and Animal Husbandry

The experiments were conducted at Tovetorp Research Station (58° 57′ N, 17° 9′ E) between 8^th^ and 23^rd^ of March 2011. Great tits were caught in mist-nets and trap-cages in the vicinity of the research station.

The birds were housed indoors individually in cages (80 x 60 x 40 cm, width x height x depth; room temperature ∼17°C). The cage floor was supplied with commercial litter and two bowls, one with water (for bathing and drinking) and one supplied with sunflower seeds. In addition, a piece of suet was attached beside one of the two perches that furnished the cage. On a daily basis, birds were offered mealworms (*Tenebrio molitor*) for enrichment, new water and replenishment of food. Thus, both water and food was supplied *ad libitum*. The indoor lighting regime was adjusted on a weekly basis to match the natural light cycle including half an hour dusk and dawn. After participating in the experiment, the birds were ringed and released at the site where they were captured.

European swallowtail (*Papilio machaon*) larvae were obtained from a laboratory population at Stockholm University. Larvae were reared singly on fresh cuttings of one of their natural host plants, parsnip (*Pastinaca sativa*) [24] in 1.0 L plastic cups. Upon eclosion, half of the butterflies were euthanized by freezing and the other half were kept in a cage and were fed a 20% sucrose solution for approximately a week whereupon they were kept in a cold room (10°C) until the experiments were conducted. Euthanized butterflies were mounted, with a pin through the thorax and their wings stretched out, on a mounting board. The mounted butterflies were dried for 10–15 days.

### (b) Experimental Procedures

**Figure 1:**
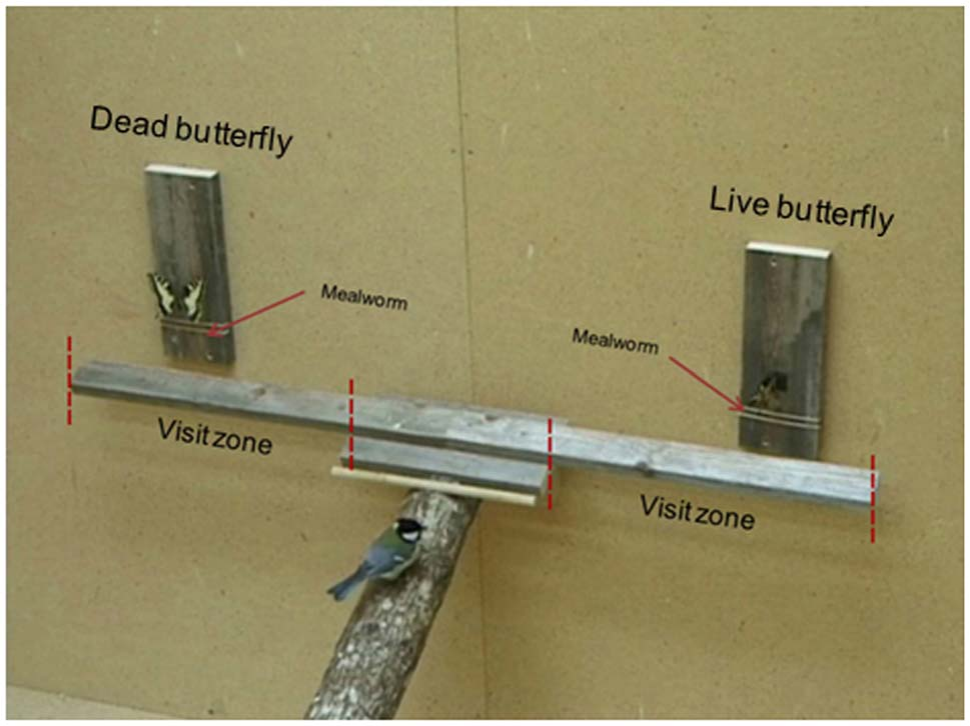
A snapshot from the video recordings on the experimental setup.

The experiments were performed in small room (2.4 x 2.3 x 2.0 m). The experimental room was furnished with a straight oak branch (1.5 m in length and 10 cm in diameter) that leaned against one of the walls. A 1.0 m plank was affixed orthogonally at the upper end of the branch. Above this plank, two smaller planks (10 x 30 cm) were mounted vertically on the wall (one at each side, 80 cm apart). A piece of rubber was glued onto the middle of each of these smaller planks and made up the position where the butterflies were presented (experimental setup: see Figure 1). The birds were encouraged to search for food on the branch by presenting a mealworm in a Petri dish that was glued onto the lower end of the branch. A bowl of water and a 1.8 m wooden perch was placed on the floor close to the lower end of the branch. The opposing wall to the setup was equipped with a one-way see-through window, and a camera (SONY DCR-V1000 E) was mounted above this window (near the ceiling) and was pointed towards the upper part of the branch to record interactions between the bird and the butterflies. The experimental room was illuminated by eight fluorescent tubes (Philips TL-D 90 Graphica Pro 36W/950).

Prior to a trial a live and a dead butterfly were placed on the two smaller planks that were attached to the wall (one at each plank). The dead butterfly was pinned at the small piece of rubber, exposing the dorsal side of the wings, and the live butterfly was placed at the small piece of rubber at the other plank. Positioning of the dead and live butterfly (right/left) was alternated between trials. A euthanized mealworm was attached below each butterfly using two stretched rubber bands. We did this for two reasons; because great tits in the lab were fed mealworms regularly, this constituted familiar and popular food; so the mealworms were placed close to the live and dead butterfly to attract the birds to the vicinity of the butterflies. Hence, even if a great tit did not attack a butterfly *per se*, the eventual hesitance of a bird to approach a butterfly, and its reaction to the deimatic display of the live butterfly, would be recorded by noting the time it took for a bird to eat the mealworm immediately below the dead, or the live, butterfly.

Furthermore, a euthanized mealworm was placed in the Petri dish at the lower end of the branch to encourage birds to search for food on the branch and the orthogonally positioned plank, from which the butterflies and the mealworms were easily accessible to the birds. The temperature was kept low (8.3°C ±1.1 (SD)) in order to prevent the butterfly from taking flight during the experiment.

A trial started when we released a great tit into the room. Each trial was allowed to proceed for 45 minutes, but was terminated earlier if the bird had taken both mealworms (and/or attacked both butterflies). Birds that had attacked both mealworms and/or butterflies were given 10 additional minutes to see if they consumed the live butterfly. We noted which butterfly that was visited first and the number of visits to each butterfly (live/dead) that were required until the mealworm or butterfly was attacked. We also noted which butterfly (or mealworm) that was attacked first and the time until the first and the second mealworm/butterfly was attacked. In trials when a butterfly or its respective mealworm was not attacked, ‘time until attack’ was given a value of 45 minutes, that is, the maximum duration of a trial. The video sequences were studied in detail and we assessed the reaction of the birds each time wing-flicking was elicited by the live butterfly; the reactions were categorised as (i) 0 =  no discernible reaction or approaching the butterfly, (ii) 1 =  flinching and/or retreating by hopping away from the butterfly and (iii) 2 =  retreating by flying away.

We also performed a palatability test of the butterflies. Six great tits were offered freeze-killed and subsequently thawed butterflies in their home cage (one to each bird) and we noted whether the butterflies were consumed and we also looked for signs of bird discomfort afterwards.

### (c) Statistical Analysis

Which butterfly (live/dead) that was (i) visited first and (ii) attacked first, was analysed using binomial exact tests. Number of visits until attack were analysed using a paired Wilcoxon signed rank test. Time until attack was analysed using a paired t-test. Time was square root transformed to meet the assumption of normality. All analyses were performed in R [25], version 2.10.1.

### (d) Ethics Statement and Permissions

Experiments described herein comply with the current laws of Sweden and the experimental procedures and housing of birds have been reviewed and approved by the regional ethical committee (Linköpings djurförsöksetiska nämnd, Dnr 11–11). Permit to keep birds was approved by the Swedish Board of Agriculture (Dnr 31–11980/10). The birds were captured with permission from the Swedish Museum of Natural History (Dnr 52-00060/2010).

## Results

Five of 32 birds never visited any of the two butterflies and will not be considered in the analyses. The 27 birds that visited at least one of the butterflies did not show any preference for visiting the dead or live butterfly first with 12 visiting the dead butterfly and 15 visiting the live butterfly first (binomial test: N = 27, P = 0.70). These 27 birds were used in all analyses unless stated otherwise. In total, 24 of 27 birds visited the dead butterfly at least once, 26 birds visited the live butterfly at least once, and 23 birds visited both the live and dead butterfly at least once.

**Figure 2:**
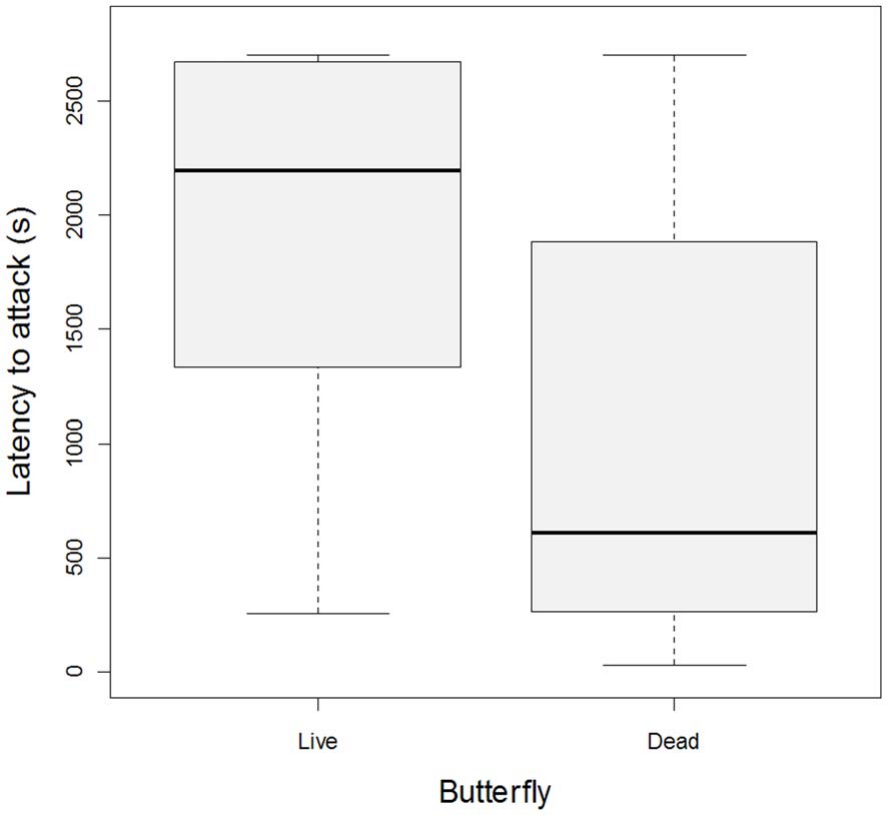
The time until the butterfly or its mealworm was attacked. Birds that did not attack either the butterfly or mealworm were given a value equalling the maximum time for the experiment, that is, 45 minutes. The boxes show median, first and third quartiles. N = 27.

Twenty-four birds attacked at least one of the butterflies (or their respective mealworm); however, more birds (N = 22) attacked the dead butterfly first (or the mealworm below it), compared to the reversed order (N = 2) (binomial test: N = 24, P = 0.0001). As a consequence, it took longer until the live butterfly (or its mealworm) was attacked (paired t-test: N = 27; df = 26; t = 5.23; P<0.0001; Figure 2). In these two analyses some of the birds did not actually attack both butterflies (or their mealworms); to assure that this did not skew our results, we repeated the analyses including only the birds that attacked the mealworm both under the living butterfly and under the dead butterfly (N = 16). These analyses returned very similar results (‘Attack order’: N_dead = _15, N_live_ = 1; binomial test: P = 0.00052; ‘Latency to attack’: median_dead_ = 497 s; Q_1_−Q_3_ = 291–731; median_live_ = 1602 s; Q_1_−Q_3_ = 1057–2342; paired t-test: N = 16; df = 15; t = 5.60; P<0.0001).

Twenty-three of 26 birds that visited the live butterfly at least once experienced at least one event of wing-flicking. In total, the 26 birds visited the live butterfly 158 times; this elicited wing-flicking in the butterfly 119 times (75.3%) whereas the butterfly remained motionless 39 times (24.7%). The birds’ reaction to the butterfly’s wing-flicking (N = 119) was either (i) retreating by instantly flying away (54.6%; see Video S1), (ii) flinching and/or escaping by hopping backwards (29.4%) or (iii) no discernible reaction or approaching the displaying butterfly (16.0%). It is noteworthy that 21 of 23 birds that experienced the butterfly’s wing-flicking reacted with showing the strongest response at least once (i.e. flew away immediately) and none of the birds showed no reaction to the butterfly’s display throughout all consecutive visits. Since there was great variation in how many times the birds experienced the butterfly’s display (range between 1 to 15 times) it is difficult to statistically address whether birds in general ceased responding to the butterfly’s display after several consecutive visits. However, it was obvious that no rapid habituation occurred; for example, the first 4 encounters generally yielded very strong responses from the birds (1^st^ wing-flicking event: N_birds_ = 23; mean response (± SE) = 1.65±0.13; 2^nd^: N = 21; mean response  = 1.62±0.15; 3^rd^: N = 16; mean response  = 1.50±0.16; 4^th^: N = 13; mean response  = 1.62±0.18; recall that reactions were scored as no discernible reaction ( = 0), flinching and/or retreating by hopping away ( = 1) or retreating by flying away ( = 2)). It should be mentioned that the mean responses appeared to be somewhat reduced after more than 4 consecutive visits, and thus suggest that a few of the birds ceased responding strongly to the display. However, the sample size of birds is drastically reduced as the number of display events increases, and, indeed, the two birds that experienced the highest number of display events (11 and 15 times respectively) actually responded by flinching and/or evading the displaying butterfly also at their five last visits which suggests that habituation was not a general phenomenon in our experiment.

Since the birds were most often strongly intimidated by the display of the live butterfly more visits were paid to live butterflies (median  = 5.0 visits; Q_1_−Q_3_ = 2.5–7.5) compared to dead butterflies (median  = 1.0 visits; Q_1_−Q_3_ = 1.0–1.0) until the birds snatched the mealworm and/or attacked the butterfly (paired Wilcoxon signed rank test: N = 27; V = 346.5; P<0.0001). This analysis was repeated incorporating only the birds that attacked both mealworms (see reasoning above) and the result was very similar (median_live_  = 6.5 visits (Q_1_−Q_3_ = 3.00–9.25), median_dead_  = 1.0 visits; Q_1_−Q_3_ = 1.0–1.25; paired Wilcoxon signed rank test: N = 16; V = 120; P = 0.00071). Although some birds were cautious when confronting also the dead butterfly, the above results reflect the modest reaction of the birds and 18 of the 23 birds that visited the dead butterfly at least once attacked the butterfly (9 birds) or its mealworm (9 birds) at the first visit which means that these birds were not intimidated by the static representation of the butterfly (see Video S2, which shows the typical behaviour when the birds visited the dead butterfly). In contrast, only 1 of 26 birds that visited the live butterfly at least once attacked the butterfly (1 bird) or its mealworm (0 birds) at the first visit and it is noteworthy that this particular butterfly remained motionless prior to the attack (18/5 vs. 1/25; Fisher’s exact test: P<0.0001). The same analysis, but considering only the attacks on the butterflies, returned a similar result (9/14 vs. 1/25; Fisher’s exact test: P = 0.0034).

In total, 9 of 27 birds eventually attacked the live butterfly. Two of these birds successfully killed the butterfly and consumed the entire body with only the wings being left and one bird attacked the butterfly (which fell to the floor) during the experiment and launched a new attack immediately subsequent to the termination of the experiment; the bird was allowed to continue its attack and consumed the butterfly as the other two birds did. The six other birds that attempted to attack the butterfly were either intimidated when the butterfly started to perform its display or dropped the butterfly on the floor and thereafter refrained from further attacks (or were again repelled by the butterfly’s display). Five of the 9 birds that attacked the live butterfly launched their attack before the butterfly had initiated its display during that specific visit (see end of Video S1), whereas 3 birds approached and attacked the butterfly although it had initiated its display prior to the attack and one bird attacked the butterfly which employed its defence concurrently with the bird’s first strike.

All six freeze-killed butterflies that were offered to the birds were consumed within an hour. As in the experiments, the birds consumed the entire butterfly body with only wing fragments being left on the cage floor. The birds did not show any signs of finding the butterflies unpalatable.

## Discussion

A majority (24 of 27) of the European swallowtail butterflies survived our experiment of being confined in a small room with a passerine predator. Furthermore, the birds were more likely to attack the dead butterfly or its mealworm before they attempted to attack the live butterfly or its mealworm. In most cases (75%) when a bird approached the live butterfly it would employ wing-flicking and this behaviour was clearly discouraging to the birds; in a majority of the visits the bird flew away instantly (55%), or flinched and/or retreated by hopping away from the butterfly (29%). Since birds typically reacted to the sudden display of the butterfly with strong evasive actions, considerably more visits were required at the live butterfly compared to at the dead butterfly until the birds attacked the butterfly or its mealworm. It is noteworthy that the current experimental setup may have led to a somewhat unnatural situation in that birds that were scared off by the butterfly’s display were constrained by the limited space of the experimental room and therefore more likely to return to the butterfly compared to what would happen during an encounter in the wild. Indeed, it is likely that a predator that has been severely intimidated from a prey animal’s startle behaviour would seek safer foraging grounds, and evidence suggests that small predators often behave like this [11]. For this reason, we argue that the most important finding of the current study was that more dead butterflies than live butterflies were attacked at the first visit and that this signifies the adaptive function of performing wing-flicking in the European swallowtail. The evasive response of the birds suggests that wing-flicking behaviour of the European swallowtail can be considered as deimatic behaviour, *sensu* Edmunds [1] and thus is involved in the butterfly’s secondary defence repertoire.

The functional mechanisms explaining why deimatic behaviour is an effective means of anti-predator defence is still largely unknown. Edmunds [1] noted that deimatic behaviours in prey either constitute honest signals of true defensive properties or capabilities, or that the signalling is a bluff, which means that prey lacks further defensive armory if the display fails to discourage the predator from attacking. Principally, wing-flicking and exposure of customarily deployed aposematic colours (black and yellow) of the swallowtail may advertise unpalatability and so could explain why the great tits were discouraged. However, the current study demonstrates that great tits offered freeze-killed and thawed butterflies devoured them without any signs of nausea; indeed, these birds had *ad libitum* access to highly preferable food such as suet strongly suggesting that the swallowtails were not only edible but rather palatable to the birds. An alternative explanation why the great tits typically refrained from attacking the live swallowtails could be that the wing-flicking display honestly informs the birds that the butterfly is elusive or by other means difficult to handle, cf. [7]. This interpretation, however, is also unlikely to apply since the birds in our experiments could easily attack the mealworm that was attached below the live butterfly regardless of whether they perceived the wing-flicking butterfly as an arduous prey to attack or not. Furthermore, strong evasive responses are not expected from the great tits if they merely conceived the swallowtails as an elusive prey. We contend that the evasive responses elicited in the great tits in our experiment are best explained by a pervasive requisite in these birds to avoid real danger. The functional efficiency of this kind of bluff defence of a palatable prey probably hinges upon three conditions concerning the deceived predator:(i) the predator must be a mesopredator and subjected to predators of its own thereby selecting for vigilance, (ii) encounters between predator and prey must occur infrequently to prohibit habituation and therefore, (iii) the predator must be a generalist [10]–[13], [26].

It stands to reason that a mesopredator’s assessment of whether an encountered item is a prey or a potential threat necessarily precedes assessment of that item’s potential noxiousness or unprofitability, which leaves the road open for the evolution of bluffing displays in palatable prey species. It is noteworthy that bluff displays are typically performed only as a secondary defence, i.e. once the prey realizes that it has been discovered by a potential predator. Hence, the discovered prey has little to lose by performing a threatening display, the efficiency of which is likely to be condition-dependent, e.g. [27].

It is interesting that the sudden wing-flicking display by the European swallowtail was quite successful as a defence against great tits, despite its absence of eyespots such as those displayed by peacock butterflies [18], [19]. Traditionally, the efficiency of eyespots on butterfly wings in intimidating predators has been believed to be due to their mimicking the eyes of the predator’s own predators (“The Eye mimicry hypothesis” [19]). The current study shows clearly however that sudden wing-flicking is strongly discouraging to small passerines even when the display does not include large eyespot and it is an open question whether the display of the swallowtail would have been even more intimidating if the wings bore large eyespots. More experiments are needed to disentangle the role of the various components that potentially add to an effective startle display. Specifically, questions that would be worthwhile to address is whether the conspicuous coloration is essential or whether the size and sudden opening of the wings explains the strong evasive responses of the birds, or whether all stimuli work in orchestration.

## Supporting Information

Video S1 **Shown are typical evasive responses by great tits when confronted with live European swallowtail butterflies that employ their wing-flicking display.**
(WMV)

Video S2 **Shown are typical modest reactions by great tits when confronted with dead, mounted specimens of the European swallowtail butterfly.**
(WMV)
